# Visualising nanoscale restructuring of a cellular membrane triggered by polyelectrolyte microcapsules†
†Electronic supplementary information (ESI) available: Fig. S1–S3. See DOI: 10.1039/c8nr03870h


**DOI:** 10.1039/c8nr03870h

**Published:** 2018-09-03

**Authors:** Yuxiu Chen, Gleb B. Sukhorukov, Pavel Novak

**Affiliations:** a School of Engineering and Materials Science , Queen Mary University of London , Mile End Road , London E1 4NS , UK . Email: p.novak@qmul.ac.uk ; Email: g.sukhorukov@qmul.ac.uk

## Abstract

Polymer-based multilayer microencapsulation technology represents one of the promising strategies for intracellular drug delivery, however, membrane processes involved in vehicle internalisation are not fully understood. Here we employed a scanning probe microscopy technique called Scanning Ion Conductance Microscopy (SICM) to study these complex processes at nanoscale resolution in real time. We were able to image topography simultaneously with local elastic modulus throughout the whole course of microcapsule internalisation in A549 cell culture without disrupting the internalisation process. The imaging revealed that capsules triggered the formation of membrane protrusions in their vicinity, which is an important but not a sufficient step towards full capsule internalisation. A crucial aspect appeared to be nanoscale restructuring of these protrusions into smooth thin layers extending over the surface of capsules. Simultaneous mapping of elastic modulus during capsule internalisation allowed monitoring the structural changes during extension of the membrane sheets over the surface of the capsule and the subsequent post-internalisation phenomenon of capsule buckling. To our knowledge these are the first experimental data capturing the interactions between the cellular membrane and microcapsules in their whole complexity with nanoscale resolution. The methodology established here has the potential to provide new insights into interactions at the interface between the nanostructured materials and cellular membrane under physiological conditions.

## Abstract 2

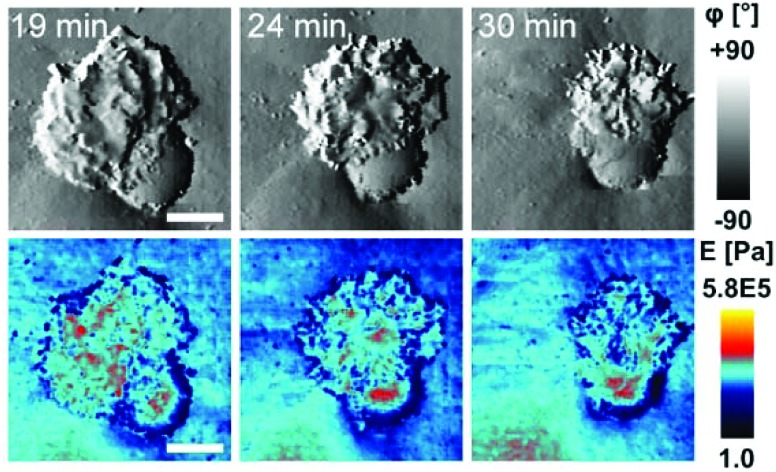
Simultaneous time-lapse imaging of topography and elastic modulus of microcapsule internalisation reveals distinct nanoscale restructuring of membrane protrusions.

## Introduction

Usability of many chemical substances with a significant potential for biomedical applications is limited by their poor solubility in water or limited stability in the physiological environment. One of the promising strategies for the therapeutic targeted delivery of these types of substances into cells and tissues is their encapsulation inside polyelectrolyte multilayer microcapsules (PMC).^1^,^2^ The successful internalisation of PMCs loaded with various macromolecules has been observed in several types of living cells,^3^,^4^ however the mechanisms of the uptake of capsules by living cells are not yet fully understood. A detailed understanding of physico-chemical and mechanical interactions between capsules and livings cells is required for specific targeting, effective delivery, and elimination of any potential toxic side effects. This has been largely limited by capabilities of available imaging techniques and the lack of specific fluorescent markers for certain types of cellular uptake. Previous studies focused mainly on studying the rate of internalisation of microcapsules at the level of cell population using conventional optical/fluorescence microscopy, confocal microscopy, and flow cytometry.^5^–^7^ These conventional fluorescence methods are known to be prone to overestimating the number of internalized capsules due to their limited capability to exclude capsules which were not fully internalized and remained attached to the cell surface.^8^ Experimental evidence with resolution high enough to resolve the fine membrane processes interacting with microcapsules has been limited to fixed samples imaged by scanning electron microscopy and transmission electron microscopy^4^ capturing randomly timed “snapshots” of what is likely to be a highly dynamic and complex interaction. Physical force interactions between the cellular membrane and capsules during internalisation were suggested to cause buckling of capsules based on indirect evidence obtained using fluorescence microscopy in live cells^9^ and separate measurements of capsule deformation under colloidal probe atomic force microscopy (AFM) outside the cellular environment.^10^,^11^ However, our knowledge of the mechanical properties of the fine membrane structures directly involved in the internalisation process or how these structures form during the internalisation is very limited, if non-existent.

Here we employ a different approach based on a high-resolution scanning probe technique called scanning ion conductance microscopy (SICM). SICM uses reduction in ionic current through the probe represented by an electrolyte-filled glass nanopipette immersed in a saline solution to detect proximity of the sample surface.^12^,^13^ This technique has been previously used for high-resolution scanning of biological samples of complexity similar to what can be expected in the case of microcapsules interacting with cells,^14^,^15^ and also for mapping mechanical properties at high resolution.^16^,^17^

## Materials and methods

### Capsule preparation

Capsules were prepared using poly(sodium 4-styrenesulfonate) (70 000) (PSS), poly(allylamine hydrochloride) (15 000) (PAH), ethylenediaminetetraacetic acid (EDTA), calcium chloride and sodium carbonate, all bought from Sigma Aldrich, UK. Polyelectrolyte microcapsules were assembled using the layer-by-layer assembly technique as described previously.^1^ Briefly, PSS and PAH were deposited onto sacrificial calcium carbonate cores with 5 μm in diameter synthesized by mixing calcium chloride and sodium bicarbonate. After 6 layers of alternate PSS and PAH were coated, the sacrificial cores were dissolved using EDTA. The capsules were then washed and re-suspended in PBS at a concentration of 10^6^ capsules per ml for future use.

### Cell culture and preparation for imaging

For live cell imaging, A549, an immortalized human lung cancerous epithelial cell line (European Collection of Cell Cultures) was cultured in high glucose DMEM (Invitrogen, UK) supplemented with 1% penicillin–streptomycin–glutamine solution (Invitrogen, UK) and 10% fetal bovine serum (Invitrogen, UK). Cells were seeded onto 35 mm glass bottom Petri-dishes FluoroDish™ with 10 mm wells (World Precision Instruments, UK) and grown into a ∼50% confluency at 37 °C, 5% CO_2_ after one to two days of incubation. The cells were then washed three times with serum-free Leibovitz's L-15 medium (Invitrogen, UK) and imaged using SICM in L-15 medium. The temperature of the medium in the bath was controlled at 37 °C by using a TC-10 temperature controller (NPI electronics, Germany) connected to an objective lens heating mantle (ALA Scientific Instruments, USA) as described previously.^18^ 100 μl of microcapsule suspension (∼10^5^ of microcapsules) was dipped into the Petri dish and the scan began once capsules started landing on the cells using an optical microscope.

### Confocal microscopy of capsule internalisation

In the experiments using confocal microscopy, the cells were dyed with CellMask orange plasma stain (Life Technologies, UK) and fixed after one-hour incubation at 37 °C with the suspension of capsules where the outermost layer consisted of green fluorescent dye FITC (ThermoFisher, UK) conjugated PAH. The sample was then imaged using a confocal microscope Leica TCS SP2. The images were analysed using Leica Application Suite.

### SICM setup

Hopping probe ion conductance microscopy was used for all topography imaging using a custom-built scanning head controlled by a SICM scanner controller (Ionscope Ltd, UK) as described previously.^14^,^15^ The scanning head consisted of a P-733.2DD XY Piezo-Nanopositioning Stage (Physik Instrumente, Germany) with a 30 μm travel range (XY movement of the sample) and P-753.21 piezo actuator (Physik Instrumente, Germany) with a 25 μm travel range (Z movement of pipette). All piezos were driven by 200 W peak power high voltage PZT amplifiers E-505 (Physik Instrumente, Germany). The XY piezo scanner incorporated into a heavy stainless-steel platform was placed onto an Eclipse TE300 Inverted Microscope (Nikon Corporation, Japan).

The pipette current was detected *via* an Axopatch 200B (Molecular Devices, USA) using a gain of 1 mV pA^–1^ and a low-pass filter setting of 2 kHz. The internal holding voltage source of the Axopatch-200B was used to supply a DC voltage of +200 mV to the pipette. Pipettes with an estimated inner diameter of 100 nm pulled from borosilicate glass of 1.0 mm outer diameter and 0.5 mm inner diameter (Sutter Instruments Co., USA) using a P-2000 laser puller (Sutter Instruments Co., USA) were used in all experiments. Localization of microcapsules and the positioning of the pipette were facilitated using optical microscopy with oil-immersion objective 100× 1.3 NA (Nikon Corp, Japan).

### SICM imaging and mapping of elastic modulus

For topography imaging, the pipette approaches until the current is reduced by a predefined amount of 0.3–0.4%. The position of the *z* dimension actuator when the current achieves this reduction is recorded as the height of the sample at this imaging point. For the measurement of elastic modulus, the pipette travels further down after reaching the first set point until the current is reduced by another 1% (to set point of 1.3–1.4%) and a second height is recorded. The difference in these two heights is used to calculate the deformation (strain) of the sample at that image point. The stress on the sample is caused by the intrinsic colloidal pressure between the cell surface and the glass tip at higher set points (>1%) which varies with the size of the tip and set point value, and is kept constant during a scan.^19^ The elastic modulus of the sample is then calculated simply as a ratio of the applied stress to the observed strain between the two set points at each imaging point using custom built image processing software, SICM Image Viewer, using the algorithm developed by Clarke *et al*.^19^ The elasticity maps have the same image resolution (effective pixel size) as topographical images, which was typically 320 nm for a 30 μm × 30 μm scan and 160 nm for a 15 μm × 15 μm scan.

### Analysis of membrane roughness and thickness

The topographical line profiles across the centre of capsules undergoing internalisation were extracted from topographical images using Gwyddion (http://gwyddion.net/) and imported into Origin 8.1 (OriginLab, USA). Ideal circle equation1
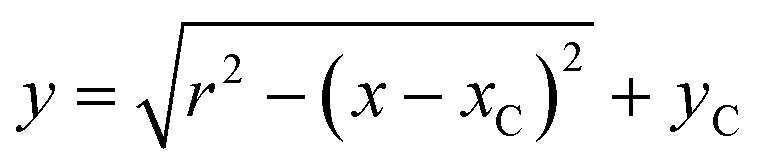
(where *r* is the capsule radius, and *x*_C_ and *y*_C_ are the coordinates of the center), representing microcapsules was fit to sections of height profiles showing the exposed capsule surface. Roughness of the membrane-coated and exposed surface of the capsules was calculated as the standard deviation of the extracted line profiles after subtracting the fit circle equation.

## Results and discussion

### Imaging internalisation of capsules using SICM

**Fig. 1:**
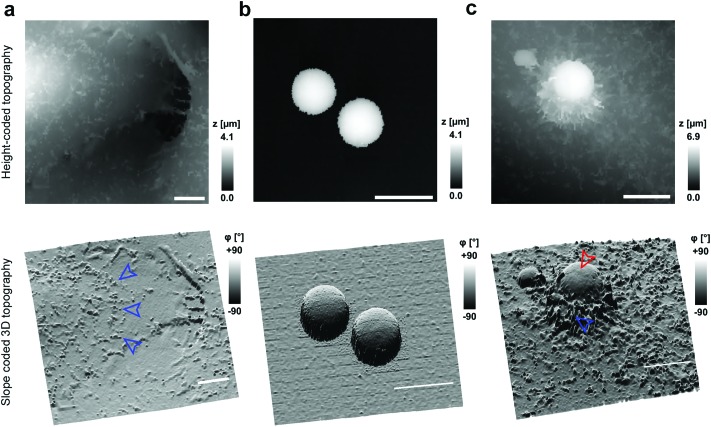
Hopping mode SICM is capable of imaging polyelectrolyte capsules in the hydrated state at high resolution. (a) Representative height-coded (top) and slope-coded (bottom) topography of the A549 cell line prior exposure to microcapsules. The slope is calculated as the first derivative of topography converted into angle *φ* representing the local slope of the topography. Note the presence of naturally occurring membrane protrusions (blue arrowheads). (b) Representative images of freshly prepared polyelectrolyte capsules in the fully hydrated state adhered to the surface of a tissue culture dish coated with PEI. Image resolution (effective pixel size) 88 nm. (c) Representative high-resolution image of the capsule (red arrowhead) approximately 15–30 minutes after landing on the membrane of the A549 cell. Image resolution (effective pixel size) 156 nm. Note the fine, finger-like membrane protrusions (blue arrowhead) accumulated around the capsule. All scale bars: 5 μm.

**Fig. 2:**
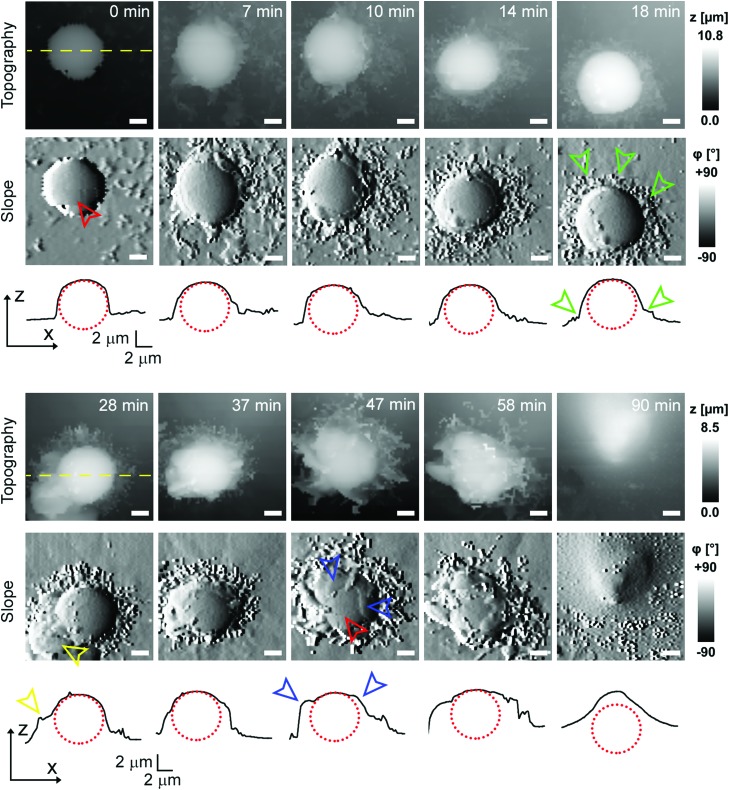
Representative internalization of the polyelectrolyte capsule displaying distinct extracellular engulfment. Time lapse imaging of topography is shown together with the corresponding slope images (first derivative of topography) and line profiles (black curves) across the centre of the capsule as indicated by the yellow dashed lines. The time 0 min marks the beginning of the recording, the exact time when the capsule landed on the cell is uncertain. The red dotted circles represent the capsule. Note how the bare capsule (red arrowhead) is gradually surrounded by fine finger-like protrusions (green arrowheads) at the bottom of the capsule, later morphing into larger membrane protrusions (yellow arrowhead), and finally how the top of the capsule gets gradually covered by thin layers of the membrane (blue arrowheads). All scale bars: 2 μm.

**Fig. 3:**
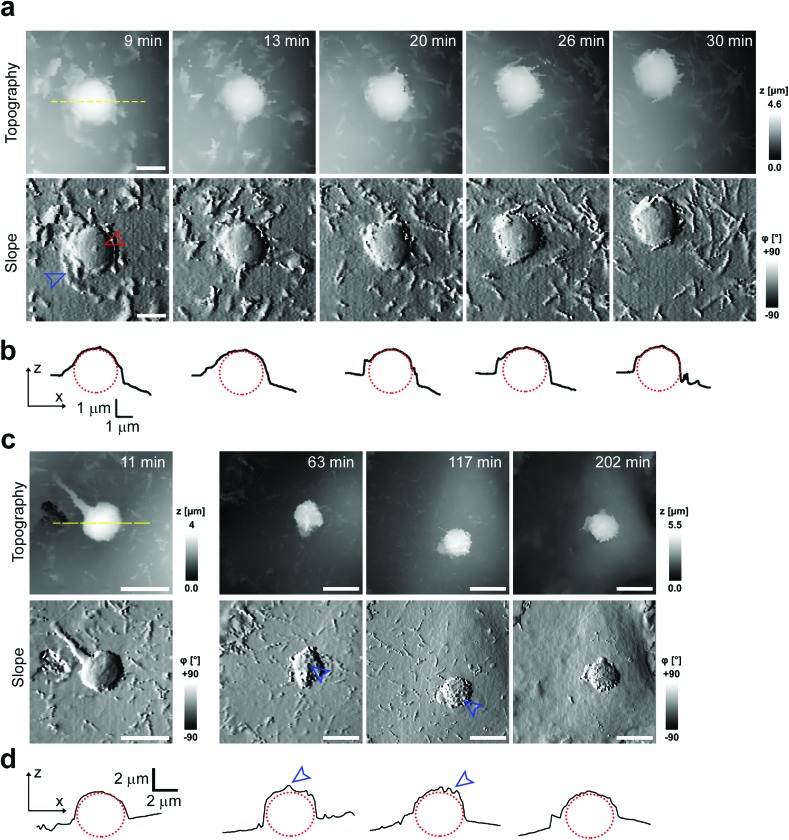
Representative time lapse SICM imaging of a “stalled” internalisation. (a) Topography and corresponding slope (first derivative of topography) images. Note that a number of protrusions (blue arrowhead) seem to be interacting with the capsule (red arrowhead), however the central area of the capsule remains almost intact. Scale bars: 2 μm. (b) Line profiles across the centre of the capsule as marked by the dashed yellow line in (a). The red dotted circle represents the capsule. No apparent sinking of the capsule was observed within the total recording time of 55 min. (c) Excerpts from a long-term time-lapse topography imaging of a stalled internalization event. Note fine protrusions (blue arrowhead) on the top of the capsule are still present 1–2 hours after the beginning of recording instead of flat, smooth sheets of the membrane observed in successful internalization events. After 202 minutes, the cell membrane around the capsule became almost completely smooth. Scale bars: 5 μm. (d) Line profiles across the centre of the capsule as indicated by the dashed yellow line in (c). The red dotted ellipse represents the capsule at given *z*–*x* scaling. Note the presence of rough protrusions on top of the capsule (blue arrowheads). No apparent sinking of the capsule into the cell visible here within 202 minutes from the beginning of recording.

To investigate cell–capsule interactions we used adenocarcinomic human alveolar epithelial cell line A549 (Fig. 1a), which may serve as a model target for drug-delivery, and has been studied before for capsule internalisation using conventional imaging techniques.^4^ The hopping mode of SICM proved to be capable of capturing freshly produced polyelectrolyte capsules adhered to the bottom of the tissue culture dish (Fig. 1b) as well as live internalisation of the polyelectrolyte capsules with high resolution (Fig. 1c). To our knowledge, these are the first high-resolution topographical images showing the capsules in their fully hydrated state, in contrast to the collapsed, dry state commonly shown previously.^9^ Note that the protrusions which are typically homogenously distributed over the membrane of A549 cells under control conditions (blue arrowheads, Fig. 1a) appear to concentrate near the base of the capsule (blue arrowhead, Fig. 1c). The complex topography of the dynamic internalisation process involving fine membrane protrusions interacting with a one order of magnitude larger capsule required a compromise between the speed and resolution of the scan. A small area of 15 μm × 15 μm could be imaged at a lateral resolution of 160 nm at the rate of one frame per 4–7 min without disrupting the internalisation. This frame rate was found to be sufficient to capture the process of capsule internalisation in the A549 cell line. We followed 27 different capsules and in 14 cases we observed complete capsule internalisation. Full internalisation occurred within 17 to 71 minutes from the beginning of the recording, with an average duration of 43.2 ± 19.3 minutes (mean ± standard deviation of the mean, Fig. S1a†). The duration of internalisation showed weak, statistically insignificant dependence on the diameter of microcapsules (*R* = 0.315, *p* > 0.05, Fig. S1b†). This was in agreement with the results obtained using confocal fluorescence imaging where 52% (17 out of 33) of capsules appeared to be internalized after one-hour incubation (Fig. S1c and d†), suggesting that SICM imaging had not negatively affected the rate of capsule internalisation. In a typical internalisation event (Fig. 2), the fine, finger-like membrane protrusions normally present on the cell first started to concentrate mainly around the bottom of the capsule (green arrowheads, Fig. 2). Some of these protrusions gradually restructured into membrane ruffles (yellow arrowhead, Fig. 2) and were followed by a number of apparently separate sheets of membranes gradually extending over the top of the capsule in a process similar to the zipper model of phagocytosis.^20^ The gradual engulfment of the capsule occurred mainly by the extension of membrane sheets over the capsule without noticeable “sinking” of the capsule into the cell until the very last stage. While the internalisation events we observed here displayed certain characteristics resembling receptor mediated phagocytosis, we would like to note here that these capsules were not modified in any way to specifically trigger the Fc or C3bi receptors previously identified as responsible for phagocytosis, and it remains unclear which, if any, receptor is involved in the processes described here. Membrane protrusions started forming within minutes from the moment of the first contact (Fig. S2†). The formation of finger-like membrane protrusions interacting with a capsule attached to the cell membrane appeared to be triggered regardless of the position of the capsule on the cell or the eventual fate of the capsule. In 13 out of the total number of 27 experiments this initial interaction between the cell membrane and the capsule appeared to “stall” in the early stages (Fig. 3) and did not progress into the development of membrane ruffles or sheets. On average, we followed these stalled events for 83 ± 46 min (*n* = 13), with the longest recording reaching 202 minutes (Fig. 3c & d) with no signs of progression into the phase where “sinking” of the capsule could be detected, or any other sign on internalisation. This suggests the formation of membrane protrusions around the capsule is a typical feature of the initial interaction between A549 cells and capsules but not a sufficient step in the process of internalisation. It appears that the important step leading to successful internalisation is restructuring of the finger-like membrane protrusions into membrane ruffles which then extend smooth sheets of the membrane over the top of the capsule. This was not observed in the cases where internalisation appeared to “stall” (Fig. 3). The existence of capsules “trapped” on the cell membrane as described here suggests that simple washing used in previous research^6^ may not be sufficient to remove capsules that have not been internalized.

### Mapping mechanical properties of capsule internalisation

**Fig. 4:**
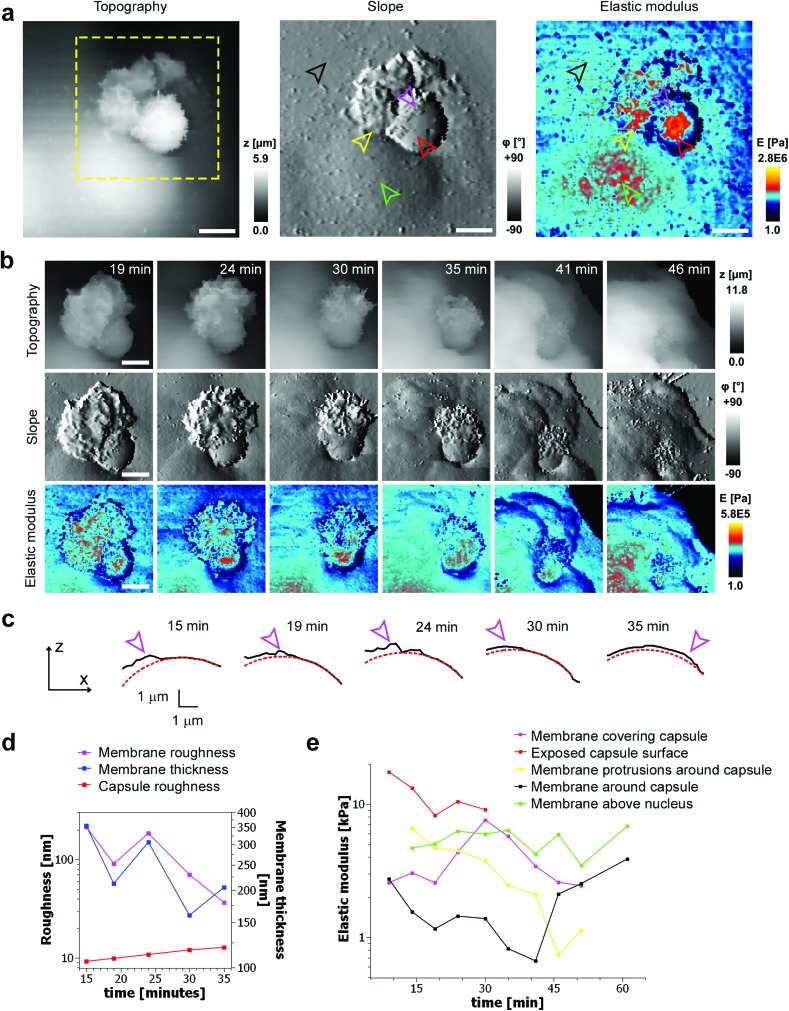
Representative time lapse imaging of mechanical properties during internalisation. (a) Topography image of 30 μm × 30 μm area showing the early stage of the internalisation together with the corresponding slope and elastic modulus images. Note the part of the capsule covered with the membrane (magenta arrowhead) displays lower elastic modulus than the exposed part (red arrowhead). Membrane protrusions (yellow arrowhead) formed in the vicinity of the capsule and also the membrane above the nucleus (green arrowhead) showed higher modulus than the membrane (black arrowhead) away from the capsule. Scale bars: 5 μm. (b) Simultaneous time lapse imaging of the topography (top row) and elastic modulus (bottom row) of the internalisation marked by a yellow dashed square in (a). Elastic modulus of the substrate was set to 1 Pa (black colour coded) for clarity. Scale bars: 5 μm. (c) Height profiles extracted from sequence in (b) showing extension of the membrane (magenta arrowhead) over the capsule surface fitted to a circle with 3.8 μm radius (red dotted line). (d) Changes in the thickness (blue line) and roughness (magenta line) of the membrane as it keeps extending over the capsule surface shown in (c). Note that roughness of the exposed capsule surface (red line) remains low and steady throughout the whole process. (e) Changes in the mean elastic modulus of the different areas of the membrane and capsule during the internalization shown in (b).

To investigate the mechanical properties of capsule internalisation we used the recently implemented method for the measurement of elastic modulus.^19^ Thanks to the low stress and minimally invasive character of the imaging using nanopipettes we were able to map changes of mechanical properties without apparent disruption of the internalisation process. The area of the cell membrane above the cell nuclei and around the nuclei displayed a mean elastic modulus of 4205 Pa ± 1284 (*n* = 9) and 1310 Pa ± 559 (*n* = 27), respectively. These values are comparable to previous observations using AFM.^21^ The mapping of elastic modulus using SICM was sensitive enough to distinguish between the bare surface of a capsule and the surface already covered by the cellular membrane (magenta & red arrowheads, Fig. 4a). The modulus of the membrane covering the capsule was similar to the modulus of the cell membrane away from the site of internalisation (black arrowhead, Fig. 4a), while the modulus of the large protrusions growing in the vicinity of the capsule (yellow arrowhead, Fig. 4a) was apparently higher suggesting higher density of actin in these protrusions. As the membrane extended over the capsule, its roughness reduced (from 214 nm to 36 nm, Fig. 4d) pointing to restructuring of the underlying actin–myosin skeleton from finger-like protrusions into smooth membrane sheets. The change in roughness was very likely due to restructuring of the membrane as the roughness of the exposed capsule surface showed only slight changes (from 9 nm to 12 nm, Fig. 4d). At its lowest thickness, the membrane sheet extending over the capsule was just 160 ± 70 nm thick (at 30 minutes, Fig. 4d), compared to 355 ± 213 nm at the beginning of the process. With the membrane sheet gradually thinning, the underlying stiff wall of the capsule could be now detected when compressing the membrane to obtain elastic modulus. This was observed as a transient increase of apparent elastic modulus of the membrane (magenta line, between 15–30 minutes, Fig. 4e) right up to the value comparable to the exposed capsule (red line, Fig. 4e), followed by a decrease back to the value comparable to the cytoplasmic membrane around the internalisation site. The transient increase in the elastic modulus reflected specifically the membrane processes occurring on the top side of the capsule as indicated by the fact that the elastic modulus of the other parts of the membrane showed distinctly different trends (Fig. 4e). The elastic modulus of membrane protrusions forming around the capsule (yellow arrowhead, Fig. 4a) simply gradually decreased as the internalisation progressed (yellow line, Fig. 4e), while the modulus of the cytoplasmic membrane around the internalisation site transiently decreased before recovering back to the original value once the internalisation was completed. The latter possibly reflects depletion of actin below the capsule as observed before during phagocytosis using fluorescence techniques.^20^ Most importantly, the elastic modulus of the membrane area above the nucleus remained relatively unchanged during the whole internalisation (green line, Fig. 4e), further supporting the claim that the observed changes in the elastic modulus described above were related to capsule internalisation.

**Fig. 5:**
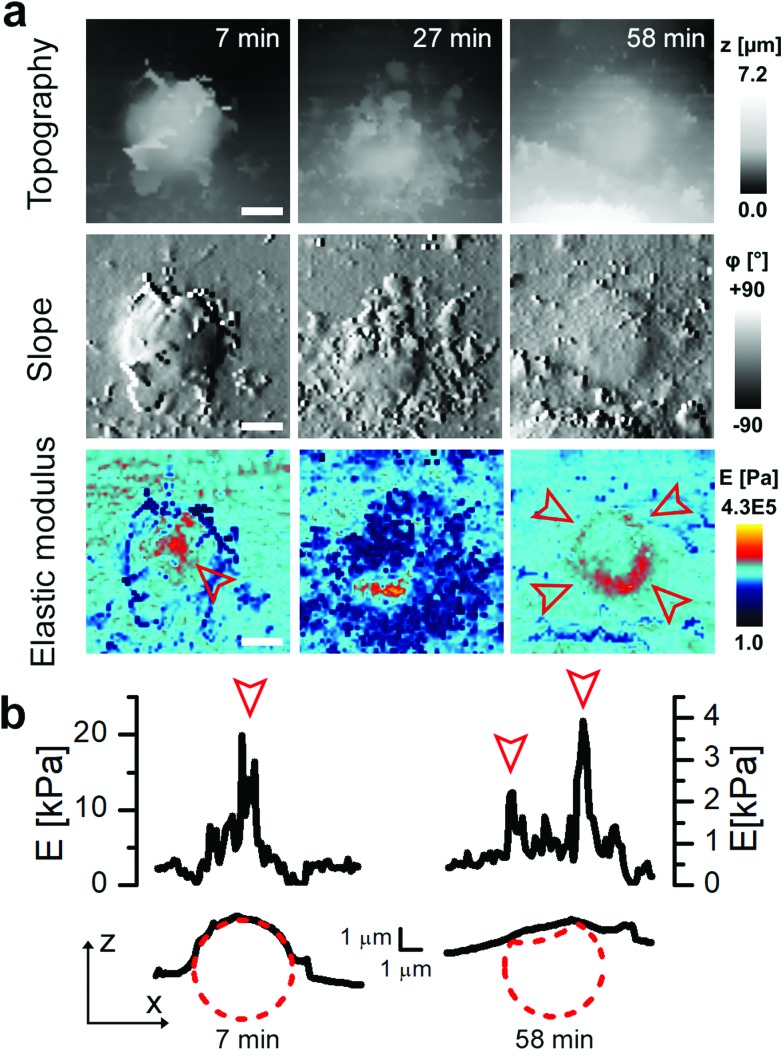
Mapping of elastic modulus can be used to study buckling of internalized microcapsules. (a) Topography, slope and elastic modulus images of 10 μm × 10 μm area showing example cases of suspected capsule buckling. Note that before the internalization the elastic modulus was higher in the centre of the capsule (red arrowhead, 7 minutes) while after full internalization it was higher at the edges creating a ring pattern (red arrowheads, 58 minutes). Scale bars: 2 μm. (b) Elastic modulus (top) and topography (bottom) profiles across the centre of the capsule shown in (a) 7 minutes (left) and 58 minutes (right) after the start of recording. The circle with a radius of 2.4 μm (red dashed line) fitted to topography data represents the capsule before and after internalization. The profile of elastic modulus observed at 58 minutes shows two peaks suggesting the collapsed centre of the capsule as illustrated by the red dotted line.

In some cases, the thickness of the membrane layer covering the microcapsule remained relatively low for a substantial amount of time following full internalisation (Fig. 5). In these cases, it was possible to visualize the microcapsule underneath the membrane using the mapping of elastic modulus for a considerable period of time and capture post-internalisation events such as buckling of the microcapsule wall (Fig. 5). Buckling of the microcapsule during internalisation under physiological conditions has been previously observed using fluorescence techniques,^9^ however the mechanical properties of the process have only been measured outside living cells.^9^–^11^,^22^ Interestingly, our observations show that the membrane protrusions surrounding and covering the capsule prior the observed buckling appear substantially softer than the capsule and also softer than the surrounding cytoplasmic membrane (Fig. 5a, at 27 minutes), and seem unlikely to be capable of applying force needed to deform the capsule. This suggests that buckling of the capsule wall may be caused by a combination of physico-chemical interactions between the top layer of the capsule wall and the membrane layer extending over the capsule rather than simple mechanical compression as suggested before.^10^

Note that the elastic modulus of the exposed capsules attached to the cell membrane estimated using our method (14 372 Pa ± 10 373 Pa, *n* = 13) is a few orders of magnitude lower than previously reported for polyelectrolyte capsules on glass coverslips (hundreds of MPa to few GPa (ref. 11)), however, it is significantly higher (*p* < 0.001) than the elastic modulus of the cell membrane just outside the internalisation site (2100 Pa ± 1202 Pa, *n* = 13). Lower elastic modulus of capsules observed in our experiments is likely due to the fact that the capsule is sitting on a soft cell with elastic modulus typically in the range of a few kPa. This explanation is supported by the fact that the measured values of elastic modulus of capsules are in a good agreement with the calculated equivalent elastic modulus of a model representing the capsule and the cell as two solid layers on top of each other exposed to the same stress (Fig. S3†). Due to very low stress imposed by the nanopipette (typically in the range of 1–100 Pa)^19^ the expected elastic modulus of capsules adhered to bare glass is beyond the measurement range of the method used here. Stress of 100 Pa would cause the capsule to compress by less than 5 pm (assuming a capsule with a diameter of 5 μm and an equivalent elastic modulus of at least 100 MPa), three orders of magnitude below the practical resolution limit of SICM.

## Conclusions

According to our knowledge the data presented in this work show the first live recordings of a complete internalisation cycle of single polyelectrolyte capsules from the viewpoint of 3D topography and mechanical properties. Previous studies using electron microscopy of fixed samples suggested the involvement of membrane protrusions, however it was not clear to what extent these protrusions are important to the process of internalisation. The high resolution topographical imaging presented here revealed that these protrusions start forming within the first few minutes of the contact between the capsule wall and cell membrane and appears to play a key role in engulfment of the capsule with the membrane right until the final stage of the internalisation when the capsule is pulled into the intracellular space. The protrusions form predominantly in the vicinity of the capsules suggesting some form of local receptor signalling similar to phagocytosis rather than the random formation of membrane ruffles in macropinocytosis. Crucially, the important step towards successful internalisation appeared to be nanoscale restructuring of the finger-like membrane protrusions into smooth sheets of the membrane extending over the capsule surface. We were able to map the changes in elastic modulus of the membrane processes involved in the internalisation with resolution high enough to resolve the gradual extension of the thin layer of the membrane over the top of the capsule and demonstrated the possibility to use measurement of elastic modulus to follow capsule internalisation even after the capsule is fully covered by the membrane. This opens possibilities for better understanding of the complex nature of membrane engulfment of a wide range of nanostructured materials under physiological conditions and improved design of these materials which would take into account their interactions with the fine membrane processes.

## Conflicts of interest

There are no conflicts to declare.

## Supplementary Material

Supplementary information
