# Supplementary material for: Visualising nanoscale restructuring of a cellular membrane triggered by polyelectrolyte microcapsules
Source: Nanoscale. 2018 Sep 3;10(35):16902–10. doi: 10.1039/c8nr03870h (PMC6137606; doi:10.1039/c8nr03870h)
Supplement: Supplementary file 1 [file NR-010-C8NR03870H-s001.pdf]

Electronic Supplementary Material (ESI) for Nanoscale.
This journal is © The Royal Society of Chemistry 2018

Supporting information

Visualising Nanoscale Restructuring of Cellular Membrane Triggered by Polyelectrolyte Microcapsules

Yuxiu Chen, Gleb B. Sukhorukov, Pavel Novak**

School of Engineering and Materials Science, Queen Mary, University of London, Mile End Road, London, E1 4NS, United Kingdom

* Correspondence to:

Pavel Novak p.novak@qmul.ac.uk, Gleb Sukhorukov g.sukhorukov@qmul.ac.uk

Figure S1. Characterizing the duration and success rate of internalization of polyelectrolyte microcapsules in A549 cell line. (a) Distribution of the duration of capsule internalization as observed using topographical

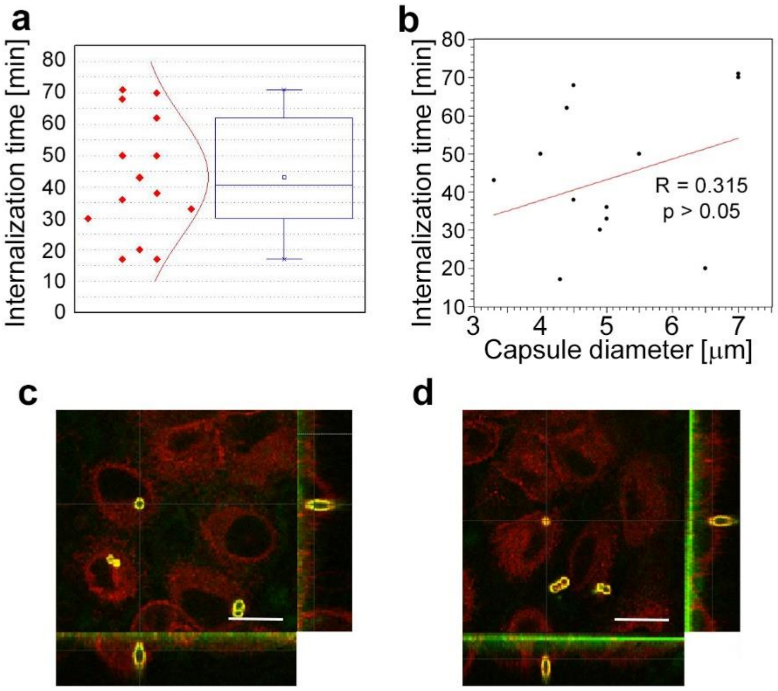

imaging with SICM. (b) The internalization time was not significantly dependent on the diameter of capsule (correlation coefficient R = 0.315, p > 0.05). (c-d) Confocal microscopy of A549 cells (in red) after one-hour incubation with microcapsules (in green) showing examples of internalized microcapsule (c) and microcapsule still residing on the cell membrane (d). Scale bars 50 µm.

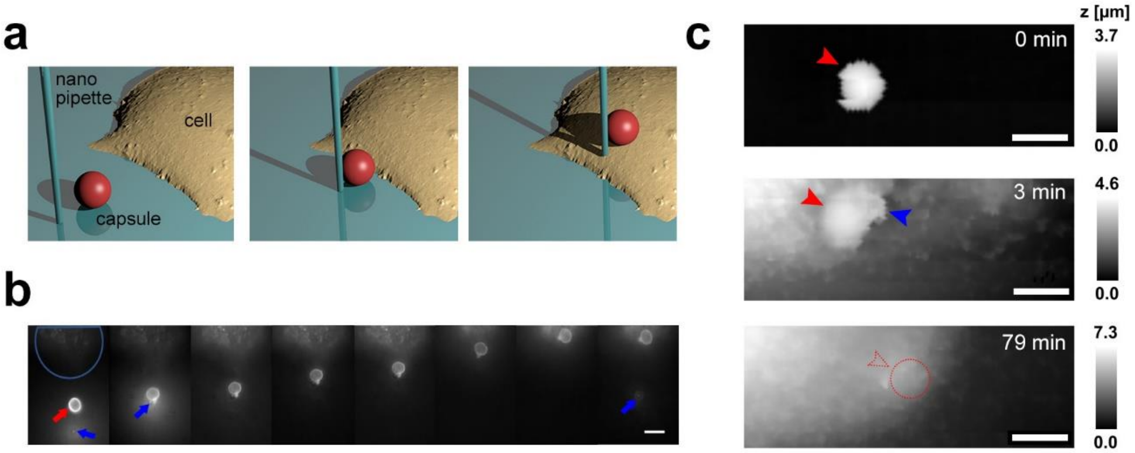

Figure S2. Membrane protrusions start forming around the capsule within minutes from the first contact. (a) In order to define the moment of first contact between the cell membrane and capsule, a nanopipette was used to transport capsule from its original landing site on a coverslip onto a nearby cell. (b) The process illustrated in (a) visualized using epifluorescence microscope. The nanopipette and the capsule are marked by blue and red arrow, respectively. The area encircled by the blue line represents nearby cell. Scale bar 5 µm. (c) Top panel, topography of a capsule (red arrowhead) which landed on the coverslip near a cell (not visible in the image). Middle panel, topography recording finished 3 minutes after capsule was moved from substrate onto the membrane of a nearby cell. Note the membrane protrusion (blue arrowhead) formed in the vicinity of the capsule. Bottom panel, topography recorded after full internalization of the capsule. The read arrowhead and the dotted red circle mark the area where the capsule was internalized. Scale bars 5 µm.

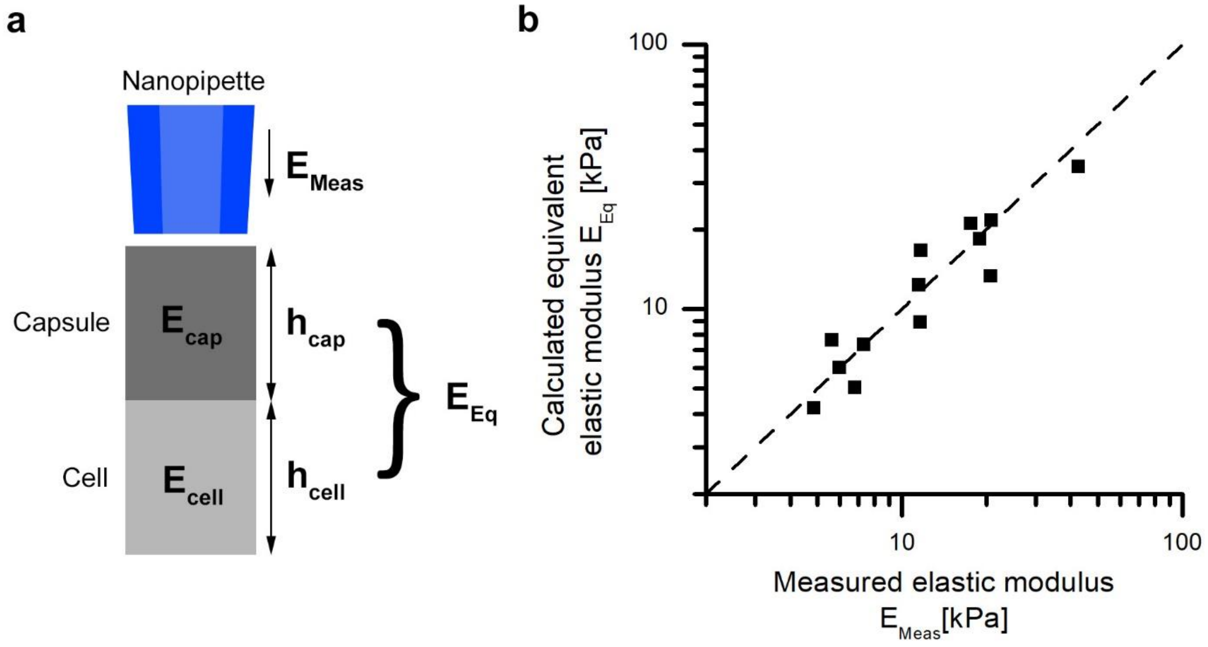

Figure S3. Measured values of elastic modulus of microcapsules on the cell membrane correspond to equivalent elastic modulus of a two-layer model. (a) Simplified model of a capsule on cell membrane. The capsule is represented by layer with elastic modulus E_{cap} and height h_{cap} which is equal to the diameter of the capsule. Cell is represented by layer with elastic modulus E_{cell} and the height h_{cell}. Compression induced by the nanopipette yields the measured elastic modulus E_{Meas}. If both layers are exposed to the same stress, then the two layers can be characterised by equivalent elastic modulus E_{Eq} calculated as: $E_{Eq} = \frac{h_{cell}+h_{cap}}{\frac{h_{cell}}{E_{cell}}+\frac{h_{cap}}{E_{cap}}}$

If the elastic modulus of the capsule is substantially higher than the elastic modulus of the cell ($E_{cap} \gg E_{cell}$), the equation for equivalent elastic modulus simplifies to: $E_{Eq} \approx E_{cell} \times \frac{h_{cell}+h_{cap}}{h_{cell}}$

(b) Comparison of the calculated equivalent elastic modulus E_{Eq} assuming the model in (a) and elastic modulus of exposed capsules sitting on cell membrane E_{Meas} measured by SICM. The dashed line represents $E_{Eq} = E_{Meas}$.
